# Urachal Adenocarcinoma: A Rare Primary Cancer Managed With FOLFOX Chemotherapy

**DOI:** 10.7759/cureus.43849

**Published:** 2023-08-21

**Authors:** Jay Patel, Augusto Villegas

**Affiliations:** 1 Digestive Disease Institute, Cleveland Clinic Foundation, Cleveland, USA; 2 Hematology and Oncology, Florida Cancer Specialists, Fleming Island, USA

**Keywords:** 5 flourouracil, urachus, bladder mass, hematuria, oxaliplatin, chemotherapy, urachal adenocarcinoma

## Abstract

Urachal adenocarcinoma (UA) represents a rare subset of bladder tumors involving a urachal remnant. Incidental gross hematuria is often the only presenting symptom, with patients often presenting late in their course, thereby imparting an overall poor prognosis. Although there are prior case reports, there is little literature reported and no standardized treatment guidelines. We report a case of a middle-aged male who presented with incidental gross hematuria after a fall. Workup indicated the presence of a calcified bladder dome mass and pathology reported a primary urachal adenocarcinoma with pelvic nodal involvement. Patient underwent surgical resection and subsequent adjuvant, systemic chemotherapy regimen with leucovorin, fluorouracil, and oxaliplatin (FOLFOX). We hope to bring greater awareness to this rare cause of bladder malignancy.

## Introduction

The urachus represents a vestigial band of musculofibrous tissue that forms early around week four of embryonic development. The urachal canal connects the allantois to the early fetal bladder [1]. The canal typically seals off around month four but can remain patent in up to one-third of adults. This is typically asymptomatic and usually only detected incidentally, and if symptomatic, it can present with congenital anomalies with leakage of urine from the umbilicus. The development of carcinomas with this process must be established [2]. 

When malignancies develop, urachal adenocarcinomas (UA) represent a rare subset, occurring in only 0.01% of all adult cancers and 0.34-0.7% of all bladder carcinomas. Although patients can have hematuria, often patients are asymptomatic and, as a result, can present late in the disease course. Its tendency for local invasion, late symptomatic presentation, and metastasis can lead to a poor prognosis, with reports indicating a mean survival of 12-24 months for locally advanced or metastatic disease [1]. Timely diagnosis is crucial, and surgical intervention remains the cornerstone for treatment. The role of adjuvant chemotherapy needs to be established [3].

## Case presentation

**Figure 1:**
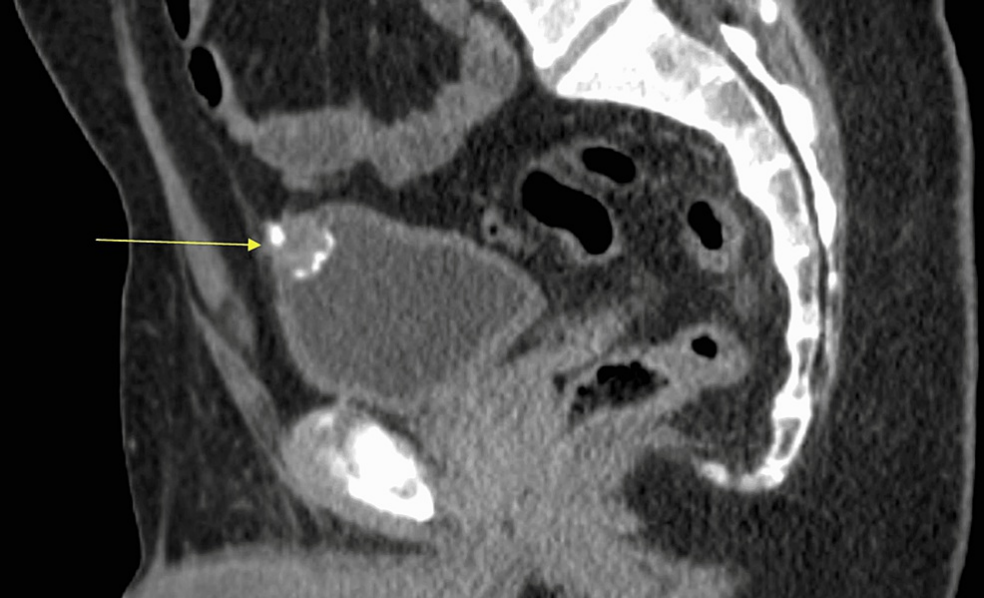
CT Urogram with automatic exposure control (AEC) indicating 1.9cm upper anterior bladder wall mass suspicious for a carcinoma.

The patient is a 40-year-old Caucasian male with a past medical history of tobacco use disorder who was referred initially to an outpatient urology office for hematuria. The patient was playing outdoors with his children when he fell and began to experience significant gross hematuria shortly afterwards, which had been persisting for around one to two weeks. The patient denied any prior history of nephrolithiasis, hematuria, or any other prior significant symptoms or hospitalizations. Vital signs were documented as being stable and the patient was not in any acute distress. Due to the persisting hematuria as well as the patient's history of smoking, urology ordered a computerized tomography (CT) urogram which indicated an enhancing lesion with calcifications at the upper anterior bladder (Figure 1).

**Figure 2:**
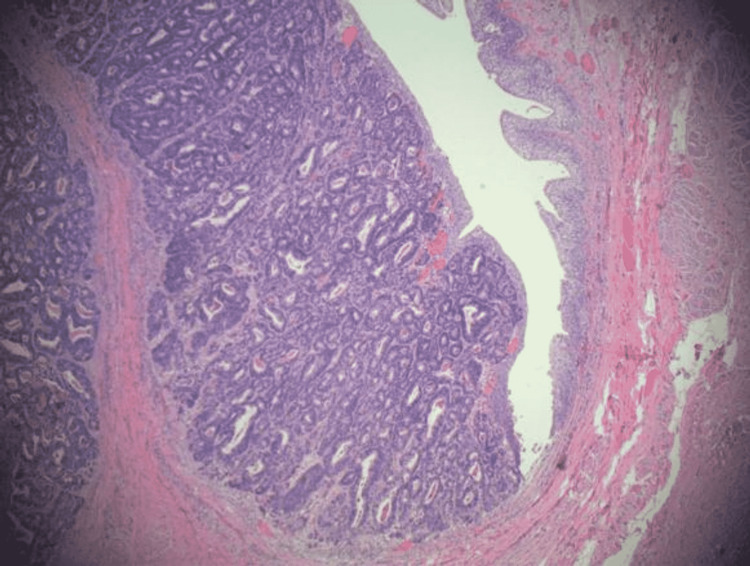
Urachal remnant tissue pathology indicating moderately differentiated adenocarcinoma with invasion of the subepithelial stroma and muscularis propria. H&E stain, 20x magnification. H&E: hematoxylin and eosin

Intraoperative cystoscopy indicated a nodular lesion at the dome of the bladder. The patient underwent laproscopic resection of the bladder mass and urachal excusion with umbillectomy. Subsequent cystogram in 10 days indicated normal findings. Pathology from the initial resection displayed a pT3B adenocarcinoma of primary urachal origin with negative margins. Analysis was positive for cluster of differentiation antigen 20 (CD20) and caudal-type homeobox transcription factor 2 (CDX2), and negative for cytokeratin 7 (CK7), p40, and GATA3 (Figure 2).

**Figure 3:**
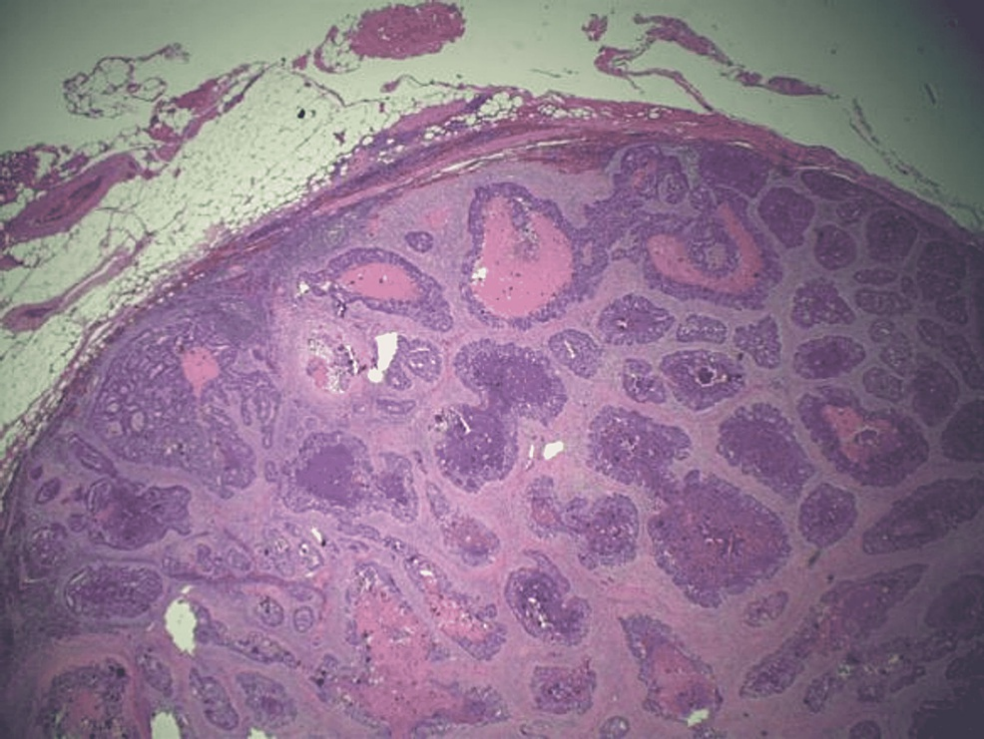
Left pelvic lymph node tissue indicating evidence of metastatic adenocarcinoma. H&E staining, 40x magnification. H&E: hematoxylin and eosin

Patient also underwent complete robotic-assisted extended resection of bilateral pelvic lymph nodes, with one left pelvic lymph node pathology that indicated metastatic adenocarcinoma (Figure 3). After discussion with the patient, oncology initiated the patient on leucovorin, fluorouracil, and oxaliplatin (FOLFOX). The patient underwent eight cycles of chemotherapy with repeat CT scans of the chest, abdomen, and pelvis and cystoscopy around six months that indicated lack of recurrent disease. 

## Discussion

​​​​​​Initially described by Begg in 1931, urachal adenocarcinoma represents a rare subset of bladder cancers, with an annual incidence of 1 in 5 million [4,5]. Malignant transformation of embryonic remnants is uncommon, and prophylactic treatment is not advocated [6]. Prior evaluation of 17 reports indicates a median age of diagnosis of 52 years, younger than non-urachal adenocarcinomas [7]. These tumors are often asymptomatic due to their extravesicular and extraperitoneal location [8]. Because of this, patients often present late in their disease course, imparting an overall five-year survival of 43%. Although patients can be asymptomatic, abdominal pain, umbilical discomfort, and discharge can occur once bladder invasion occurs. Hematuria is the most common presenting symptom, with prior reports indicating a 17-fold increase in malignancy if present [1]. Because of its rare occurrence, there have been no large, randomized, prospective trials evaluating urachal cancers, and therefore there are limited evidence-based guidelines on disease management [8]. 

Imaging can include ultrasound, which often portrays a midline soft tissue mass with mixed echogenicity and possibly calcifications. CT allows for the evaluation of local staging and metastatic involvement. Usually, a midline mass is appreciated with superior bladder dome involvement and adjacency to the abdominal wall. Peripheral calcifications can be seen and are possibly pathognomonic for urachal adenocarcinoma [4]. Distinguishing it from urothelial cancer, bladder wall invasion can be seen in up to 92% of adenocarcinomas, with 48% of cases indicating distant metastatic involvement [1]. Imaging is necessary to distinguish between urachal and non-urachal carcinomas, as no specific symptoms can differentiate [3]. A visible mass can be identified via cystoscopy in 80% of patients, while urine cytology is positive in only 38%, often negative because of the extravesicular presence of the tumor [9]. 

Pre-operative sampling can be challenging and not always feasible. A transurethral resection (TUR) can cause bladder rupture and is technically complex. In addition, TUR's reported negative predictive value is as low as 50% [10]. When imaging is inconclusive, a percutaneous sampling approach can be utilized to differentiate between benign and malignant masses [11]. However, in most circumstances, clinical suspicion with imaging findings can preclude the necessity of initial sampling while proceeding directly to surgical resection [10]. 

Various diagnostic guidelines have been proposed, but no established and widely-accepted diagnostic criteria exist within the medical community. The World Health Organization (WHO) 2016 classification includes criteria of a tumor localized in the dome and anterior wall of the bladder, cancer localized within the epicenter of the bladder wall, the absence of cystitis/glandularis beyond the bladder dome and anterior wall, the absence of urothelial neoplasia in the bladder, and the absence of primary tumor elsewhere [5]. Of note, the 2022 classification does not mention any particular update regarding urachal tumors. The Monroe Dunaway Anderson Cancer Criteria (MDACC) includes two primary and four supportive criteria. The two main criteria are the midline location of the tumor and sharp demarcation between the tumor and normal surface epithelium. Supportive criteria include the presence of enteric histology, the absence of urothelial dysplasia, the absence of cystitis cystica, and the absence of primary adenocarcinoma of another origin. It should be noted, however, that all of the prior criteria are not always expressed within patients and that a greater degree of clinical suspicion correlated with imaging analysis should drive diagnosis and subsequent management [1]. 

The most commonly cited staging system is the Sheldon staging. Direct extension to the abdominal wall, peritoneum, or other viscera, or the bladder is stage III disease. Regional node metastasis is considered stage IVA disease, while distant metastasis is classified as stage IVB disease [7]. Alternative staging systems proposed by the Mayo Clinic and TNM classification have been proposed, and prior reports have seen both as having similar effectiveness in predicting outcomes and mortality [9,12].

Various immunohistochemical markers have been proposed, including beta-catenin, CK7, CDX2, and CK20. Prior reports indicate similarities between colorectal and urachal adenocarcinomas regarding molecular analysis [13,14]. A comprehensive biomarker review indicated that CK20 and CDX2 had a 97% and 90% positivity rate for UA, respectively, as was seen in our patient [6]. Although similarities exist between urachal, gastric, and ovarian-origin adenocarcinomas, CK7 tends to be negative in gastric signet cell or colonic cancers, whereas it is positive in urachal cancer [15]. However, further studies are needed to assist in diagnosing UA via immunohistochemistry. Embryologically, there are no similarities regarding urachal and colonic development to suggest marker similarities.

Molecular analysis of UA has revealed common molecular lesions within various pathways. A study of 70 patients with urachal cancer revealed Kirsten rat sarcoma virus (KRAS) mutations in 21% of patients, BRAF in 4%, neuroblastoma ras viral oncogene homolog (NRAS) in 1%, and phosphatidylinositol-4,5-bisphosphate 3-kinase catalytic subunit alpha (PIK3CA) in 4%. Epidermal growth factor (EGFR) and ERBB2 amplifications were found in 5% and 2% of cases respectively [14]. A separate report of 22 patients with UA found KRAS mutations in 6%, BRAF in 18%, and NRAS in 5% of cases but did not report EGFR or PIK3CA mutations in any samples [13]. 

Regarding treatment, surgery remains the gold standard for the management of UA. Typically, resection of the umbilicus, urachus, radical/partial cystectomy, and complete bilateral pelvic lymphadenectomy is recommended for patients [7]. Negative surgical margins are the most crucial prognostic predictor [1]. A study of 50 patients by Herr et al. found that en bloc resection of urachal cancer and urachus combined with partial cystectomy cured 70% of patients with locally advanced cancer [16]. 

The necessity of complete bilateral pelvic node dissection remains controversial. While the prognostic efficacy of pelvic node dissection regarding overall survival has been reported [17], studies suggest that it also does not improve overall survival and carries an increased complication rate. Pelvic node positivity can also be as low as 17% [18]. Complete bilateral pelvic node dissection is encouraged to assist with staging [19]. 

The role of adjuvant or neoadjuvant chemotherapy has yet to be thoroughly established. The lack of extensive randomized trials has precluded consensus guidelines [7]. Having more scarcity of data, the role of neoadjuvant therapy in these patients is often based on expert opinion [20]. Reports suggest that neoadjuvant therapy may benefit node-positive disease [21]. The efficacy of chemotherapy has been recently recognized in decreasing cancer-specific and overall mortality, particularly in younger cohorts with metastatic disease [22]. The selection of protocols to date has primarily been presented in case reports and retrospective studies, and a definitive prospective analysis of the use of chemotherapy for UA has yet to be done. A few reports have presented the adjuvant use in metastatic patients of MVAC (methotrexate, vinblastine, adriamycin, and cisplatin), CMV (cisplatin, methotrexate, and cisplatin), FOLFIRI (fluorouracil, leucovorin, irinotecan), Gem-FLP (gemcitabine, fluorouracil, leucovorin, and cisplatin), and FOLFOX [23-26]. Fluorouracil and oxaliplatin-containing regimens are proposed based on their practical use in the management of colonic adenocarcinoma [5,14]. Because of similarities to colon cancer regarding carcinoembryonic antigen (CEA) elevations, immunohistochemistry, and tumor mucin production, FOLFOX and FOLFIRI regimens are often utilized [27,28]. A meta-analysis reported the superiority of 5-FU-based regimens compared with cisplatin-based regimens [25].

Urachal cancers are not radiosensitive, and therefore radiotherapy is seldom utilized. In a prior study of over 400 patients with UA, only 10% of patients received radiotherapy [29]. It has been occasionally utilized for locally inoperable disease or positive margins, however strong evidence is lacking to suggest improved outcomes [21]. Expert guidelines recommend that radiotherapy can be considered in patients unfit for surgery, however benefits are unclear [8]. 

Over 20% of patients have evidence of metastasis at presentation, with upwards of 59% developing metastasis at some point during disease progression. The most common sites are bone, lung, peritoneum, and liver. Chemotherapy remains the mainstay of metastatic management. Resection of single, slow-growing organ metastasis may be considered. With the advent of targeted molecular therapy, genetic and molecular mutations may be utilized for a specialized therapeutic approach [19]. Case reports have highlighted using EGFR inhibitors such as gefitinib or cetuximab [7].

Our patient had presented with gross hematuria in the setting of tobacco use, which was concerning for genitourinary malignancy. CT imaging had evidence of an anterior mass at the dome of the bladder with calcifications. Per prior management recommendations, the patient underwent resection of the bladder lesion, urachus, umbilicus, and bilateral pelvic lymph nodes. Pathology would indicate an adenocarcinoma of urachal origin with negative margins. Due to the prior success of adenocarcinomas with FOLFOX therapy, the decision was made for the patient to commence multiple rounds of chemotherapy. Guidelines regarding follow-up testing for urachal tumors are not clearly defined. Subsequent CT imaging and cystoscopy indicated a lack of recurrence.

## Conclusions

Primary urachal adenocarcinoma is a rare and potentially aggressive form of cancer arising from a urachal remnant. It can often present at very advanced stages, leading to a poor prognosis. Although there are no consensus diagnostic criteria, imaging and subsequent histopathological analysis are vital to appropriate diagnosis. Reported cases to date have utilized urological surgical resection with subsequent chemotherapy. Further studies as needed to standardize diagnostic and treatment protocols. We hope to bring greater awareness to this rare subset of urological malignancies.
